# Deep Learning Models for Stress Analysis in University Students: A Sudoku-Based Study

**DOI:** 10.3390/s23136099

**Published:** 2023-07-02

**Authors:** Qicheng Chen, Boon Giin Lee

**Affiliations:** 1 School of Computer Science, University of Nottingham Ningbo China, Ningbo 315100, China; harrison15120@outlook.com; 2 Nottingham Ningbo China Beacons of Excellence Research and Innovation Institute, School of Computer Science, University of Nottingham Ningbo China, Ningbo 315100, China

**Keywords:** deep learning, signal processing, stress detection, wearable sensing

## Abstract

Due to the phenomenon of “involution” in China, the current generation of college and university students are experiencing escalating levels of stress, both academically and within their families. Extensive research has shown a strong correlation between heightened stress levels and overall well-being decline. Therefore, monitoring students’ stress levels is crucial for improving their well-being in educational institutions and at home. Previous studies have primarily focused on recognizing emotions and detecting stress using physiological signals like ECG and EEG. However, these studies often relied on video clips to induce various emotional states, which may not be suitable for university students who already face additional stress to excel academically. In this study, a series of experiments were conducted to evaluate students’ stress levels by engaging them in playing Sudoku games under different distracting conditions. The collected physiological signals, including PPG, ECG, and EEG, were analyzed using enhanced models such as LRCN and self-supervised CNN to assess stress levels. The outcomes were compared with participants’ self-reported stress levels after the experiments. The findings demonstrate that the enhanced models presented in this study exhibit a high level of proficiency in assessing stress levels. Notably, when subjects were presented with Sudoku-solving tasks accompanied by noisy or discordant audio, the models achieved an impressive accuracy rate of 95.13% and an F1-score of 93.72%. Additionally, when subjects engaged in Sudoku-solving activities with another individual monitoring the process, the models achieved a commendable accuracy rate of 97.76% and an F1-score of 96.67%. Finally, under comforting conditions, the models achieved an exceptional accuracy rate of 98.78% with an F1-score of 95.39%.

## 1. Introduction

In this current generation, college and university students encounter a multitude of stressors, particularly in China, where the concept of “involution” gained prevalence around 2020. Academic-related stress has emerged as a significant source of anxiety for Chinese students, encompassing pressures to attain top grades, worries about receiving lower scores, concerns regarding the availability of internship opportunities compared to their peers, and anxieties about securing graduate study prospects.

Involution, also known as “Nei Juan” in Chinese pinyin, refers to situations in work and study where individuals put in extra effort, but that effort does not yield a proportional outcome [1]. For instance, the process of securing a graduate study opportunity in a university has become more challenging. In the past, students with relatively high grade point averages (GPAs) would be admitted based on limited quotas. However, admission offices now consider additional factors such as extracurricular activities and published academic papers. Consequently, students are compelled to invest extra effort in internships, extracurricular activities, and academic research to enhance their chances of admission. This intensifies the competition among students and contributes to a growing sense of stress. A survey conducted by [1] examined 1100 Chinese students and revealed that only 3% of middle school students get a full 8 h of sleep daily, while 19.5% sleep for less than 4 to 5 h. An influential article [2] written by a Chinese mother narrated her struggles to provide her child with top educational opportunities. Despite earning a monthly salary of RMB 30,000, which is considered relatively high in China, it proved insufficient due to the pressure to enroll her daughter in numerous extracurricular classes in order to excel in all exams, even if those classes covered material beyond the scope of the regular school curriculum. The combined pressures from schools, society, and even families have given rise to a highly stressed generation.

Research has indicated that high levels of stress are linked to lower levels of well-being and reduced quality of life. Prolonged exposure to stress can result in severe mental health issues such as anxiety and depression [3]. A survey involving 5551 students [4] found that anxiety is negatively correlated with academic performance, meaning that students with lower anxiety levels tend to have higher GPAs than those with moderate or high levels of anxiety. Furthermore, depression and anxiety can lead to suicide, which is the second most common cause of death among college and university students. According to a report by [5], approximately 1100 students out of 100,000 commit suicide each year. Thus, monitoring stress levels can be extremely beneficial for universities and families in supporting students’ academic performance and enhancing their overall well-being.

## 2. Related Work

Wagh et al. [6] employed Support Vector Machine (SVM), k-Nearest Neighbor (kNN), and Decision Tree (DT) algorithms to classify positive, neutral, and negative emotions using time and time-frequency domain features extracted from various channels of electroencephalogram (EEG) data. The classifiers were trained on the SJTU emotion EEG dataset (SEED), resulting in an accuracy of 72.46% for DT and 60.19% for kNN. Vijayakumar et al. [7] developed a 1D convolutional neural network (CNN) to classify arousal, valence, and liking based on peripheral physiological signals, including blood volume pressure (BVP), horizontal electrooculogram (hEOG), vertical electrooculogram (vEOG), trapezius electromyogram (tEMG), zygomaticus electromyogram (zEMG), respiration rate (RSP), and skin temperature (SKT) data. The CNN model achieved accuracies of 77.03% for arousal, 68.75% for valence, and 74.68% for liking using time and frequency domain features. Miao et al. [8] proposed a parallel spatial-temporal 3D deep residual learning framework called MFBPST-3D-DRLF for emotion recognition using EEG signals. This framework utilized multiple frequency bands (delta, theta, alpha, beta, gamma) of EEG signals to generate a 3D representation of features, which were then trained using a 3D deep residual CNN model. It achieved a classification accuracy of 96.67% on the SEED dataset (positive, neutral, and negative emotions) and 88.21% on the SEED-IV dataset (happy, fear, sad, and neutral).

Montero Quispe et al. [9] employed a novel self-supervised learning approach for emotion recognition, consisting of two stages: self-supervised pre-training and emotion recognition model training. In the pre-training stage, the model learned to recognize six signal variants generated by applying noise, scaling, negation, flipping, permuting, and time warping to the original data. This approach aimed to capture the main characteristics of the data. Results showed that self-supervised learning outperformed fully supervised learning methods in classifying arousal and valence using EEG and electrocardiogram (ECG) data. Tang et al. [10] conducted experiments on emotion recognition using EEG data with a proposed model called Spatial-Temporal Information Learning Network (STILN), which achieved an accuracy of 68.31% for arousal and 67.52% for valence. Choi et al. [11] proposed an attention-LRCN model that reduced motion artifacts in collected photoplethysmography (PPG) data. By combining the attention module with a baseline model and utilizing frequency domain features of PPG as inputs, the proposed model achieved an accuracy of 97.11%. Li et al. [12] developed a 1D convolutional neural network and a multi-layer perceptron to enhance the accuracy of stress detection using the WESAD dataset. They achieved exceptional results, with an accuracy rate of 99.80% for binary classification and an accuracy rate of 99.55% for 3-class classification. Arsalan et al. [13] focused on detecting stress during public speaking activities by collecting EEG, Galvanic Skin Response (GSR), and PPG data. They extracted frequency domain and time domain features and employed support vector machines (SVMs) with radial basis function. Their approach achieved a stress classification accuracy of 96.25% and an impressive F1-score of 95.99%. Han et al. [14] gathered and extracted features from ECG, PPG, and GSR, achieving an accuracy of 94.55% using 10-fold cross-validation and 81.82% in a real-world setting with kNN. Rastgoo et al. [15] developed a stress detection system that dynamically monitors driver stress during driving. They incorporated multiple data sources such as ECG, steering wheel, and weather conditions to predict the driver’s stress level. Their approach, combining CNN and long short-term memory (LSTM) networks, achieved an accuracy of 92.80% and an F1-score of 94.56%.

In general, biosignals such as PPG, ECG, and EEG signals have demonstrated their utility in analyzing both physical health status and mental states, including emotions, concentration, and stress. Several public datasets, such as AMIGOS [12], DREAMER [13], SWELL (Smart Reasoning for Well-being at Home and at Work) [14], SEED [15], DEAP [16], and WESAD [17], have been curated specifically for research purposes and contain carefully designed experiments to induce stress in participants while collecting these biosignals. EEG, ECG, and GSR signals were obtained from sets of videos in the AMIGOS dataset. Similarly, the DREAMER dataset and SEED dataset also employed film clips to evoke positive, neutral, and negative emotions, gathering participants’ ECG and EEG signals. The DEAP dataset utilized music videos to elicit emotions across different arousal–valence quadrants, capturing EEG and peripheral signals. The WESAD dataset utilized video clips and the Trier Social Stress Test as stimuli, collecting peripheral signals from participants. In the SWELL dataset, participants were exposed to a range of stressors, including time pressure, within a genuine office environment. They were requested to engage in activities such as report writing and giving presentations while their ECG data were systematically gathered. These publicly available datasets have been adapted for a wide range of research focusing on emotion recognition and stress detection. Meanwhile, deep learning methods, including CNN and LSTM, yielded higher accuracies compared to traditional machine learning methods such as SVM and DT.

The majority of public datasets currently available for developing stress detection algorithms utilize video and audio stimuli. The SWELL dataset has published a stress dataset whose data are collected under real-life office scenarios. However, there is no existing stress dataset found that collects data in a school context. Students are often studying and engaging in challenging problem-solving tasks, resulting in physiological signal changes that differ greatly from those observed during passive video viewing. To address this issue, this study devised a stress induction protocol that combined different levels of problem-solving (Sudoku game at medium and hard difficulty levels) with environmental stimuli such as videos and audio, creating a context in which students were required to solve problems amidst various distractions.

## 3. Materials and Methods

### 3.1. Experiment Design

In order to replicate a stress-inducing environment, participants were tasked with completing multiple Sudoku games under different scenarios, including both noisy and noise-free conditions. Throughout the experiments, various physiological data, including ECG, PPG, and EEG, were collected from the participants using wearable sensors. Following each Sudoku game, participants were asked to assess their own stress levels by completing a questionnaire. All participants were healthy students from the campus who volunteered for the experiment by filling out a registration form posted on the university’s forum. The registration form included a series of questions, such as their medical history related to heart diseases, favorite music, and music that caused discomfort. Students with heart-related conditions were excluded from participating in the experiment. The registration form was created using Wenjuanxing [18], a platform that facilitates the design of questionnaires, exams, voting systems, and rating forms.

A total of 30 participants, consisting of 6 males and 24 females, with a mean age of 20.4, were recruited for the study. Among the participants, 7 were from the Faculty of Science and Engineering (FOSE), 12 were from the Nottingham University Business School (NUBS), and 11 were from the Faculty of Humanity and Social Science (FHSS). In terms of academic status, there were 15 sophomore students, 4 junior students, 8 senior students, and 3 Ph.D. students, as shown in Table 1. Upon registration, participants were informed of the experiment’s location and schedule based on their availability. Each experimental session involved only one participant and took place in a small meeting room equipped with one desk, a couple of chairs, and a television with a functioning speaker.

The participants were given a brief introduction to the purpose of the experiment and were asked to sign a consent form and an information sheet if they agreed to participate before the experiment began. As the experiment involved solving Sudoku puzzles, the subjects were briefed on the basic rules of Sudoku. An iPad device running a Sudoku app that generated puzzles with the highest difficulty level was used. Following this, the subjects were fitted with sensors to collect physiological data. The experiment commenced only after confirming that the data receiver terminal was functioning correctly.

For each experiment, the participants were tasked with solving three Sudoku puzzles within a time limit of 15 min. The scenarios for each experiment were as follows:

- Scenario 1: The participant was left alone in the room, solving Sudoku puzzles while being exposed to horror or discordant audio, such as white noise, and watching horror videos, such as zombie movies.
- Scenario 2: No music or videos were played during this scenario. Instead, a person was present in the room observing the participant while they solved the Sudoku puzzles.
- Scenario 3: The participant was left alone in the room, solving Sudoku puzzles while being exposed to comforting audio and videos, such as sounds of birds, waterfalls, and rainfall.

The participants were divided into two study groups. The first group consisted of 15 participants who were assigned to solve Sudoku puzzles with a “medium” difficulty level. The second group also had 15 participants, but they were assigned to solve Sudoku puzzles with a “hard” difficulty level. This division was made to investigate the impact of Sudoku difficulty on changes in stress levels. At the end of each trial, participants were asked to indicate their stress levels using the re-designed questionnaire. This questionnaire was designed using Wenjuanxing and was based on the self-report approach proposed by Li et al. [15]. The questionnaire was adapted and revised for this study. The stress levels were assessed on a 3-point scale, where scores of 0 to 4 indicated relaxation or little stress, scores of 5 to 7 indicated medium stress, and scores of 8 indicated high stress. In order to enhance the level of stress and motivation during the Sudoku puzzle completion, incentives in the form of prizes were provided to the participants. The participants had the opportunity to win a prize for each Sudoku puzzle if they were able to successfully complete it within the given time limit of 15 min. Additionally, if a participant successfully completed all the Sudoku puzzles within the designated time frame, they were eligible to receive an additional prize. In total, there were four prizes available to be won.

### 3.2. Data Collection

**Figure 1:**
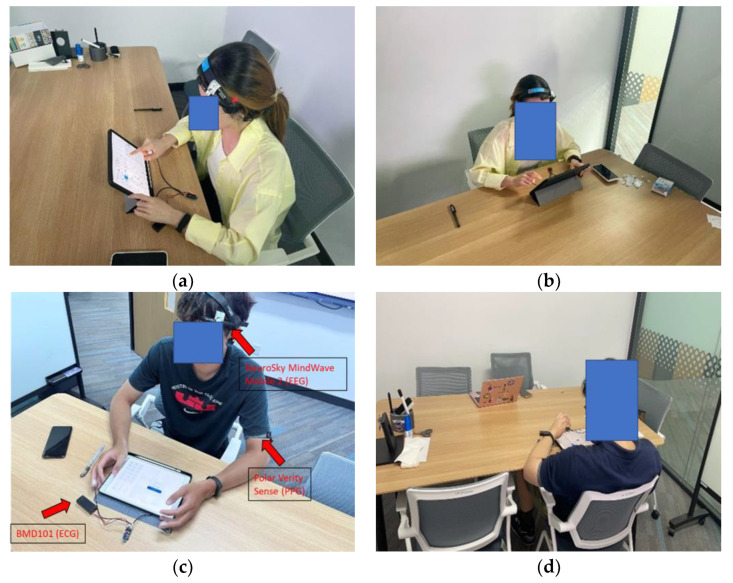
The experiment was designed as a solitary activity, with participants (**a**–**d**) solving Sudoku puzzles in a designated room. Sensors were attached to the participants during the experiment to collect data.

The data collection process involved using three separate healthcare devices to gather PPG, ECG, and EEG data from the participants, as shown in Figure 1. PPG data were collected using the Polar Verity Sense (PVS) [19], ECG data were collected using the BMD101 device [20], and EEG data were collected using NeuroSky’s MindWave Mobile 2 (MV2) [21]. The sampling rates for data collection were set at 55 Hz for PVS, 512 Hz for BMD101, and 512 Hz for MV2. It is important to note that data collection did not occur during the participant’s self-reporting periods.

To capture the signals emitted by BMD101, PVS, and MV2 devices, three distinct programs were employed. The BMD101 package [20] offered a starting program designed specifically for acquiring the ECG signal transmitted by BMD101. Additionally, PVS provided an SDK example for capturing the PPG signal [20]. To collect the EEG signals transmitted by MV2, a ThinkGear socket [21] developed with Node.js on GitHub was utilized. The program for receiving ECG and EEG signals was executed on Windows 11, while the program for capturing the PPG signal ran on an Android platform. All of these programs recorded the raw data in text files.

### 3.3. Data Pre-Processing

**Figure 2:**
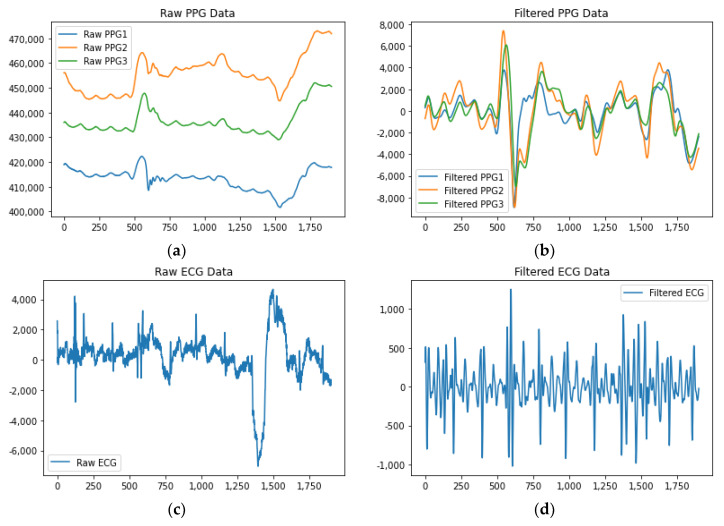

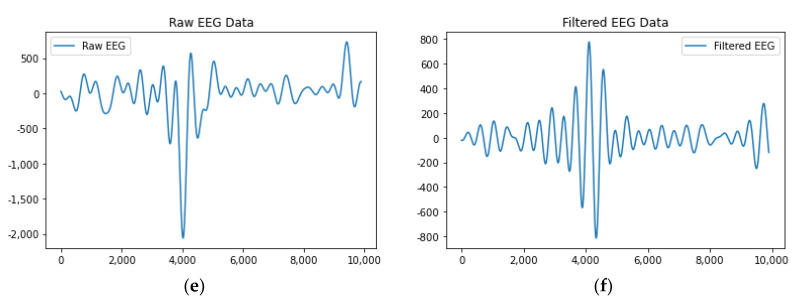
Collected (**a**) raw PPG, (**b**) filtered PPG, (**c**) raw ECG, (**d**) filtered ECG, (**e**) raw EEG, and (**f**) filtered EEG data in this study. The PPG1, PPG2, and PPG3 signals represent the green, red, and infrared light captured by the sensor, respectively. Following the application of filtering techniques, both motion-related noise in PPG and noise in ECG were effectively reduced. However, there were no discernible changes observed in the EEG signal.

**Figure 3:**
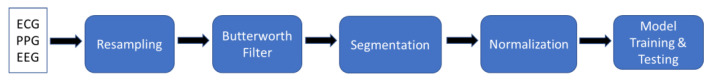
Training pipeline of a model.

**Figure 4:**
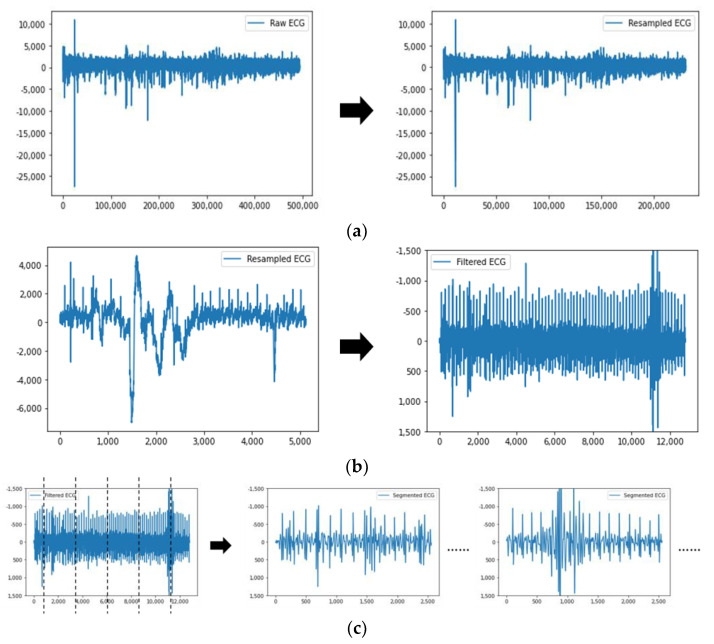

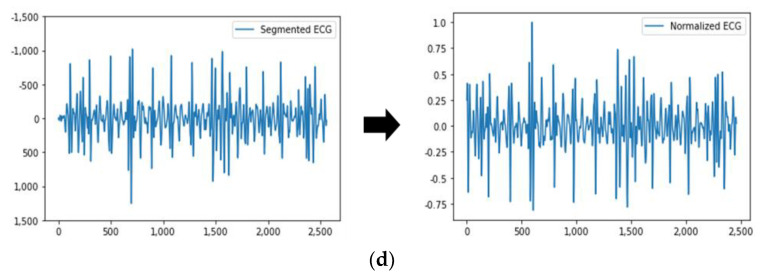
Showcase of data preprocessing procedures for a specific sample of collected ECG data. These steps include (**a**) down-sampling the ECG from 494,033 data points to 230,400 data points, (**b**) employing a Butterworth filter, (**c**) segmenting the data, and (**d**) normalizing the data.

The raw data obtained from the experiment had varying sampling rates, which posed challenges when inputting the data into the classification models without preprocessing. Additionally, there were certain inevitable recording errors during the data collection process. For example, the start time of PPG data collection may have been slightly delayed or advanced compared to the other two types of data, resulting in a few missing data points. Furthermore, there were uncontrollable variables in the experiment, such as instances where participants completed the Sudoku puzzle in less than 15 min, requiring the experiment to be ended earlier. To address these issues, all the data were resampled to a uniform rate of 256 Hz using a resampling method. This resulted in a total of 230,400 data points for each experiment. Subsequently, a Butterworth band-pass filter was applied to remove noise from the data. For PPG data, a low-cut frequency of 0.5 Hz and a high-cut frequency of 5 Hz were utilized. The raw PPG1, PPG2, and PPG3 signals corresponded to the sensor’s green, red, and infrared light sources, respectively. For ECG data, the low-cut and high-cut frequencies were set at 5 Hz and 15 Hz, respectively. As for EEG data, a low-cut frequency of 0.1 Hz and a high-cut frequency of 15 Hz were employed. For the segmentation step, a sliding window approach with a duration of 10 s and no overlap was employed on the 15 min raw data. This segmentation divided the original 230,400 data points into 90 segments, with each segment containing 2560 data points. Figure 2 depicts the unprocessed and filtered PPG, ECG, and EEG data. The training pipeline for a model is outlined in Figure 3. On the other hand, Figure 4 illustrates the process of incorporating a collected sample of ECG data, indicating the various steps involved.

### 3.4. Models Training

#### 3.4.1. StressNeXt

**Figure 5:**
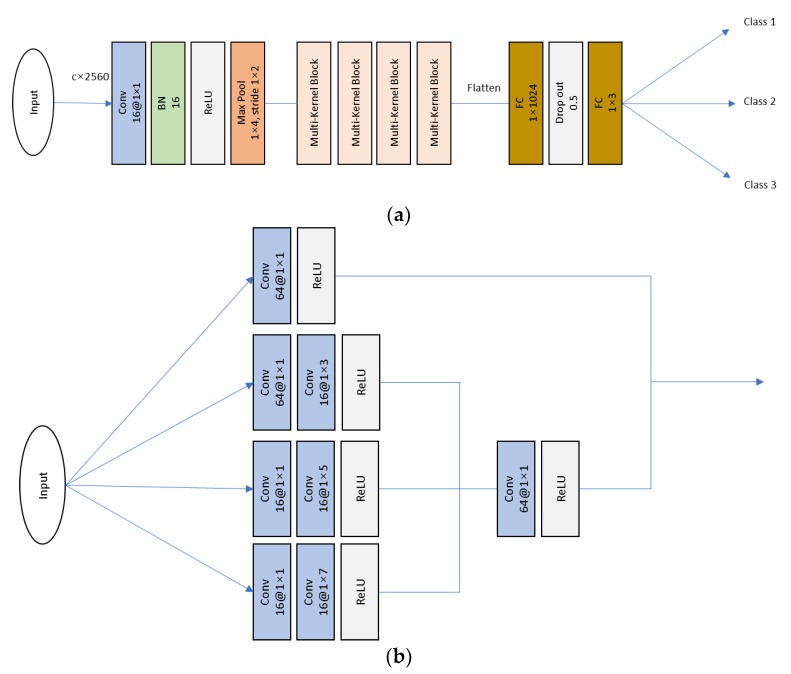
StressNeXt (**a**) model architecture and its (**b**) multi-kernel block design.

In the StressNeXt model [22], the parameter quantity is initially reduced using a 1 × 1 convolutional block. The reduced parameters are then passed through four consecutive multi-kernel blocks. Each multi-kernel block comprises multiple convolutional layers with different sizes, namely 1 × 1, 1 × 3, 1 × 5, and 1 × 7. Within the multi-kernel block, the input signal is simultaneously processed by these convolutional layers, and instead of concatenating the results at the end, similar to the Inception network [23], the feature maps obtained from these layers are combined by addition. The specific architecture of the StressNeXt model is depicted in Figure 5. 

#### 3.4.2. LRCN

**Figure 6:**
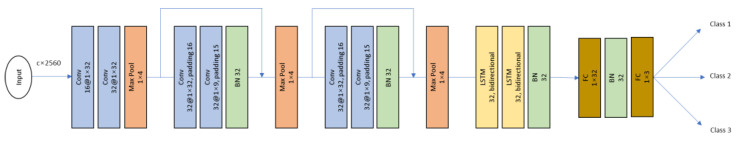
The LRCN architecture consists of 1D convolutional layers followed by ReLU activation.

The attention-LRCN (Long-term Recurrent Convolutional Network) model [14] was originally designed to take the Short-Term Fourier Transform (STFT) of signals as input, which consists of 2 dimensions corresponding to frequency and time. However, in this study, only 1 dimension is used as input. Therefore, the baseline of the attention-LRCN was taken and adapted to perform classification tasks on 1D input signals from the dataset. The model begins with two 1D convolutional layers and a max pooling layer to reduce computation complexity. Next, two residual blocks are applied, each separated by a max pooling layer that further reduces the feature maps. After the residual blocks, two LSTM layers are used to extract time-domain features from the signals and perform the classification task. Figure 6 shows the architecture of the LRCN model.

#### 3.4.3. Self-Supervised CNN

**Figure 7:**
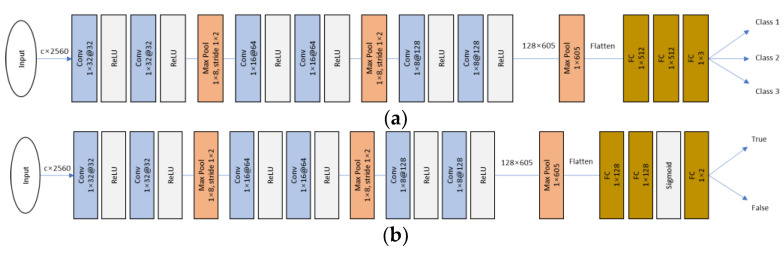
Self-supervised CNN architecture for (**a**) pretrain and (**b**) classifier.

**Figure 8:**
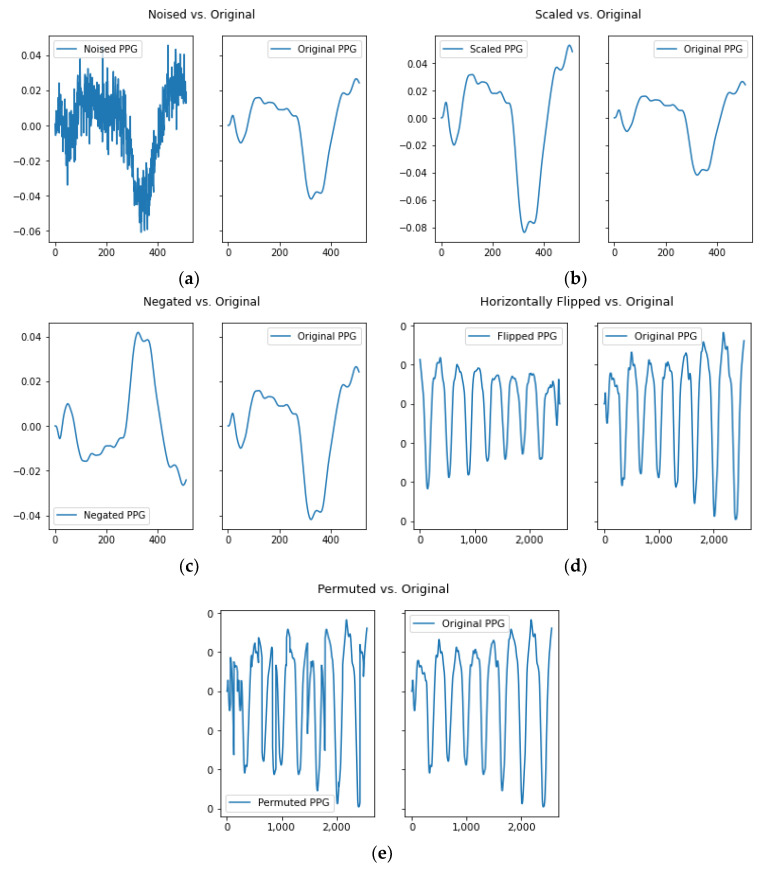
Transformed PPG vs. original PPG: (**a**) noised PPG vs. original PPG (**b**) scaled PPG vs. original PPG (**c**) negated PPG vs. original PPG (**d**) horizontally flipped PPG vs. original PPG (**e**) permuted PPG vs. original PPG.

The process of self-supervised learning consists of two stages as illustrated in Figure 7. In the first stage, known as pretraining, a self-supervised CNN [9] is trained to recognize whether an input is a transformed version of the original signals. Various transformations are applied to the signals, including adding noise, scaling, negating, horizontally flipping, and permuting the signal. However, in this study, the time-warped transformation was excluded. Before applying these transformations, the data are segmented and normalized, as shown in Figure 4. For example, Gaussian noise with parameters μ=0 and σ=0.01 is added to the signal, the signal is scaled by a factor of 1.1, and signal permutation involves randomly selecting 20 pieces of length 1/4 of the sampling rate (64 data points) and swapping them to create a permuted version of the original signal. Figure 8 displays both the transformed PPG signal and the original signal as examples for reference. After the pretraining stage, the shared layers (convolutional layers) of the self-supervised CNN are reused for classification tasks.

The shared layer of the self-supervised CNN is composed of three convolutional blocks. Each block consists of two 1D convolutional layers and is followed by a max pooling operation. In the first convolutional block, there are 32 filters with a filter size of 32. The second block has 64 filters with a size of 16, and the third block uses 128 filters with a size of 8. The max pooling layers in each block have a size of 8 and a stride of 2. After passing through the shared layer, the feature maps have dimensions of 128 × 605. These feature maps are then inputted into a global max pooling layer, resulting in an output size of 128 × 1.

#### 3.4.4. Training Parameters

The models’ performance was evaluated using k-fold stratified cross-validation. This approach ensures that each split contains all class labels rather than having one-fold with labels from only one class. The value of k was set to 3 for this training. The optimizer used was Adam, with a learning rate of 0.001. During the training of the models, the total number of epochs was set to 300, while for the self-supervised CNN pretraining stage, it was set to 150 epochs. The validation accuracy of each epoch and F1-score were averaged and returned as the performance for each fold. After completing the cross-validation, the performance of each fold was averaged again to obtain the final model performance.

The models were developed using the PyTorch framework and executed on an HP Shadow Elf 7 laptop running Windows 11. The laptop was equipped with an NVIDIA GeForce RTX 3060 Laptop GPU, an Intel(R) Core (TM) i7-11800H CPU operating at 2.30 GHz, and 16 GB of RAM.

## 4. Results and Discussion

Various signal combinations, such as PPG, ECG, EEG, PPG + ECG, PPG + EEG, ECG + EEG, and PPG + ECG + EEG, were explored to determine the most effective signals for stress detection. The performance of the models was evaluated using accuracy and F1-score as metrics. Accuracy represents the percentage of correctly classified data in relation to the entire dataset and serves as a straightforward measure of performance which is represented as follows:

$$accuracy=\frac{1}{n}\sum \left(p_{i}==y_{i}\right)$$

where $p_{i}$ is the predicted label of the *ith* sample, and $y_{i}$ is the true label of the *ith* sample. If $p_{i}$ is the same as $y_{i}$,$\;p_{i}==y_{i}$, it is 1, otherwise, it is 0. F1-score can be interpreted as a harmonic means of precision and recall,

$$F1=2\times \frac{precision\times recall}{precision+recall}$$

where precision assesses the classifier’s capacity to avoid mislabeling negative samples as positive, while recall evaluates the classifier’s ability to identify all positive samples, with the equations as follows:

$$precision=\frac{TP}{TP+FP}$$

$$recall=\frac{TP}{TP+FN}$$

where *TP* is true positive, *FP* is false positive, and *FN* is false negative.

### 4.1. Scenario-Based Self-Reporting Stress Analysis

**Figure 9:**
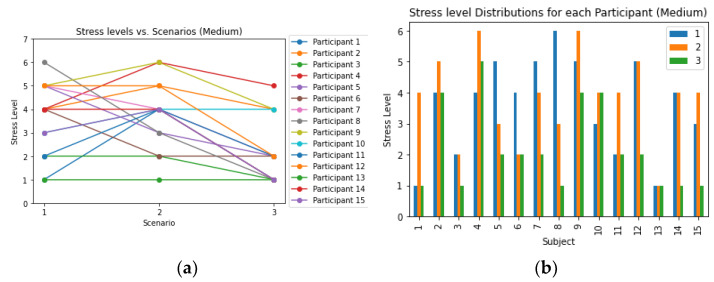
(**a**) Relationship between stress levels and scenarios, as well as the (**b**) distribution of stress levels for each participant in the medium-level Sudoku where the legend represented the scenarios.

**Figure 10:**
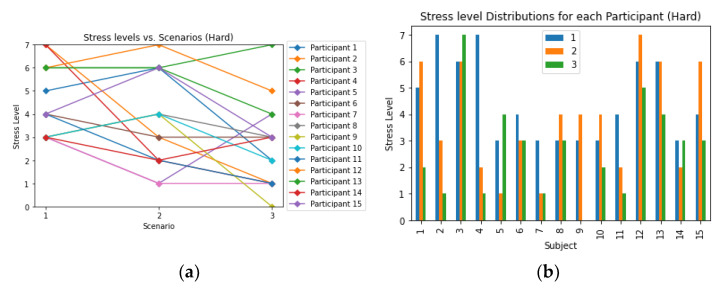
(**a**) Relationship between stress levels and scenarios, as well as the (**b**) distribution of stress levels for each participant in the hard level Sudoku where the legend represented the scenarios.

Figure 9 and Figure 10 presented the changes in stress levels across different scenarios while participants solved “medium” and “hard” levels of Sudoku puzzles, respectively. The results indicated that a majority of participants experienced an increase in stress levels in scenario 1, which involved noisy and horror audio/videos. About half of the participants (13 out of 30) reported higher stress levels in scenario 2, where they were monitored by another person. Conversely, in scenario 3, which included comforting audio and video, most participants (22 out of 30) reported a decrease in stress levels. When comparing scenario 3 to scenario 1, 20 out of 30 participants demonstrated a decrease in stress levels in the final scenario. Additionally, in scenario 1, participants solving the “hard” Sudoku puzzles tended to experience higher levels of stress compared to those solving the “medium” Sudoku puzzles. Similarly, in scenario 3, participants solving the “medium” Sudoku puzzles showed a reduction in stress levels compared to those solving the “hard” Sudoku puzzles.

### 4.2. Classifiers Evaluation

Table 2 displays the accuracy and F1-score results for different models and combinations of biosignals used in the classification. The results revealed that the LRCN model demonstrated the highest effectiveness in predicting stress levels when utilizing ECG data, achieving an average accuracy of 93.42% and an F1-score of 88.11%. Other models also exhibited high levels of accuracy and F1-score, with StressNeXt and self-supervised CNN achieving the best performance when combining ECG and EEG signals. Notably, the utilization of EEG alone resulted in the lowest accuracy and F1-score across all models. This observation could potentially be attributed to participants’ suboptimal reduction of movements during the experiments, as proposed by [24,25]. The presence of muscle noise might have compromised the effectiveness of the collected EEG data in this study.

Table 3 displays the accuracy and F1-score outcomes for the various model and biosignal combinations utilized in the stress level classification under different experimental scenarios. The findings suggest that the StressNeXt model was most successful in predicting stress levels in scenario 3, while the LRCN model was the most effective in scenarios 1 and 2. ECG was found to be the most effective signal in analyzing stress levels, with all models achieving around 95% accuracy and 90% F1-score when utilizing ECG data. On the other hand, EEG was found to be the least effective signal for detecting stress levels, with all models yielding the lowest accuracies and F1-scores, less than 70% in scenarios 1 and 2 and 90% in scenario 3. In addition, it can be observed that all models were able to predict stress levels rather effectively in scenario 3, with accuracy and F1-score reaching approximately 90% for all signal combinations.

Table 4 displays a comparison of the difficulty levels of Sudoku for each model. The accuracy rates and F1-scores of the StressNeXt model were slightly higher in the “medium” level Sudoku sample group compared to the “hard” level Sudoku sample group. Conversely, the other two models exhibited slightly lower accuracy rates and F1 scores in the “medium” level Sudoku sample group compared to the “hard” level Sudoku sample group. However, these differences were not found to be statistically significant.

**Figure 11:**
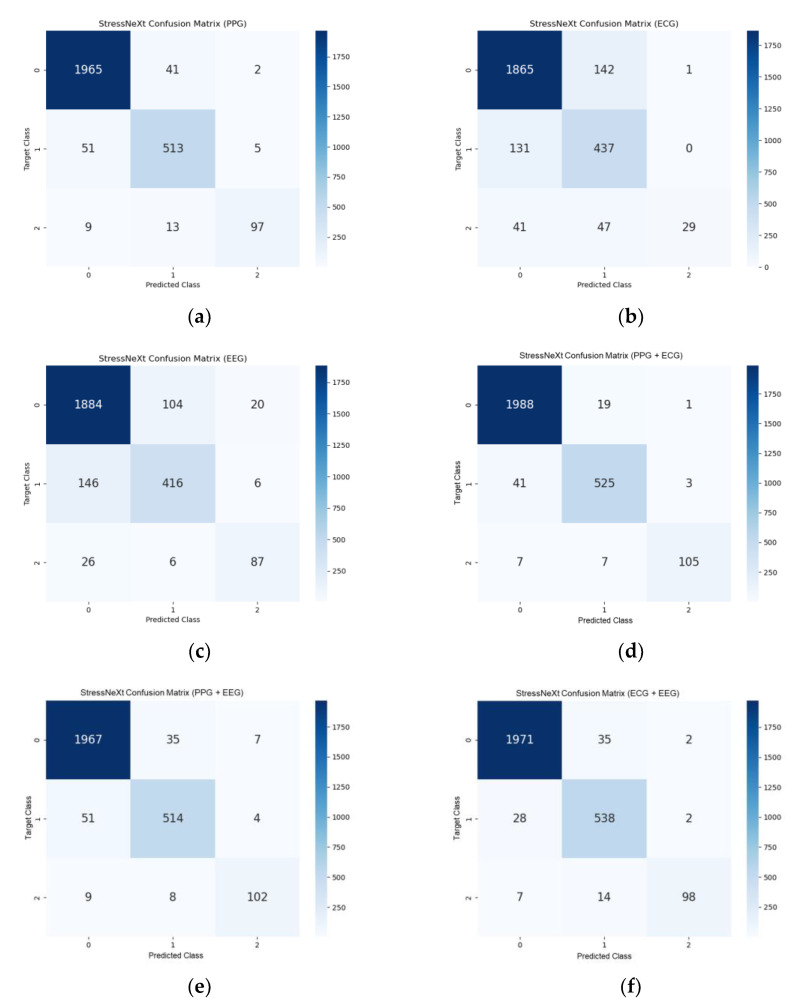

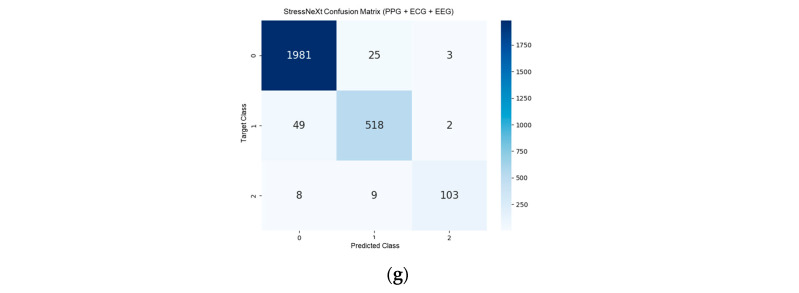
StressNeXt confusion matrix for (**a**) PPG as input, (**b**) ECG as input, (**c**) EEG as input, (**d**) combined PPG and ECG as input, (**e**) combined PPG and EEG as input, (**f**) combined ECG and EEG as input, (**g**) combined PPG, ECG, and EEG as input.

**Figure 12:**
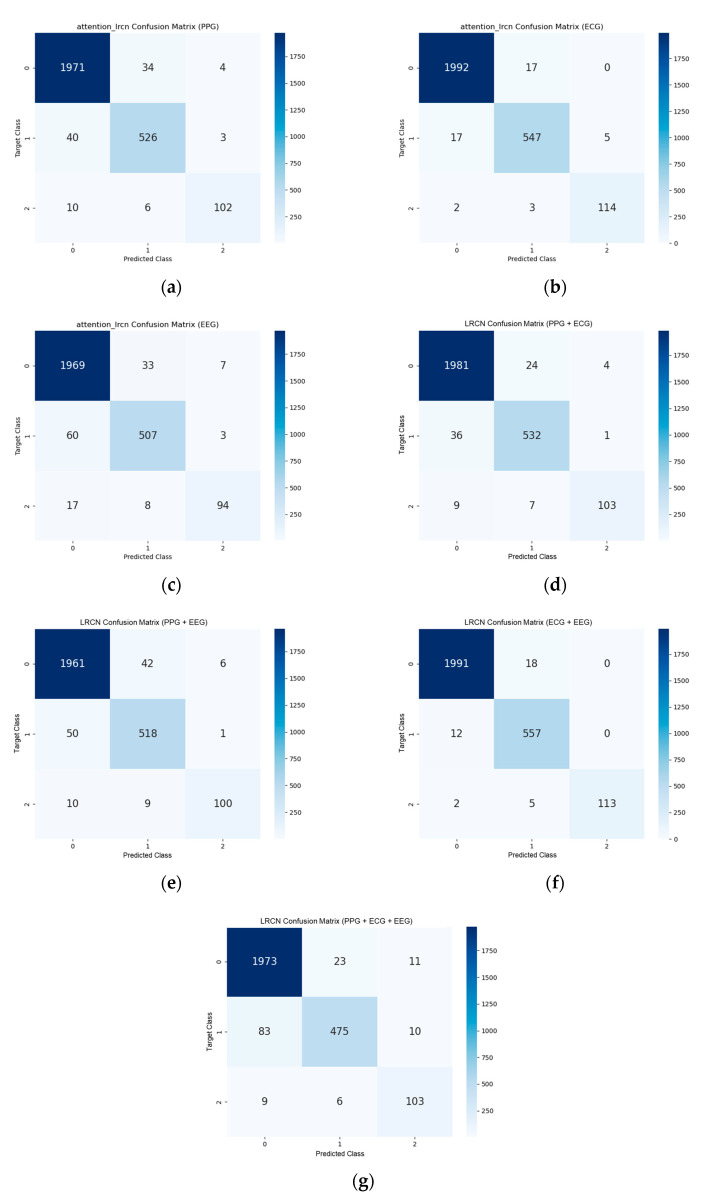
LRCN confusion matrix for (**a**) PPG as input, (**b**) ECG as input, (**c**) EEG as input, (**d**) combined PPG and ECG as input, (**e**) combined PPG and EEG as input, (**f**) combined ECG and EEG as input, (**g**) combined PPG, ECG, and EEG as input.

**Figure 13:**
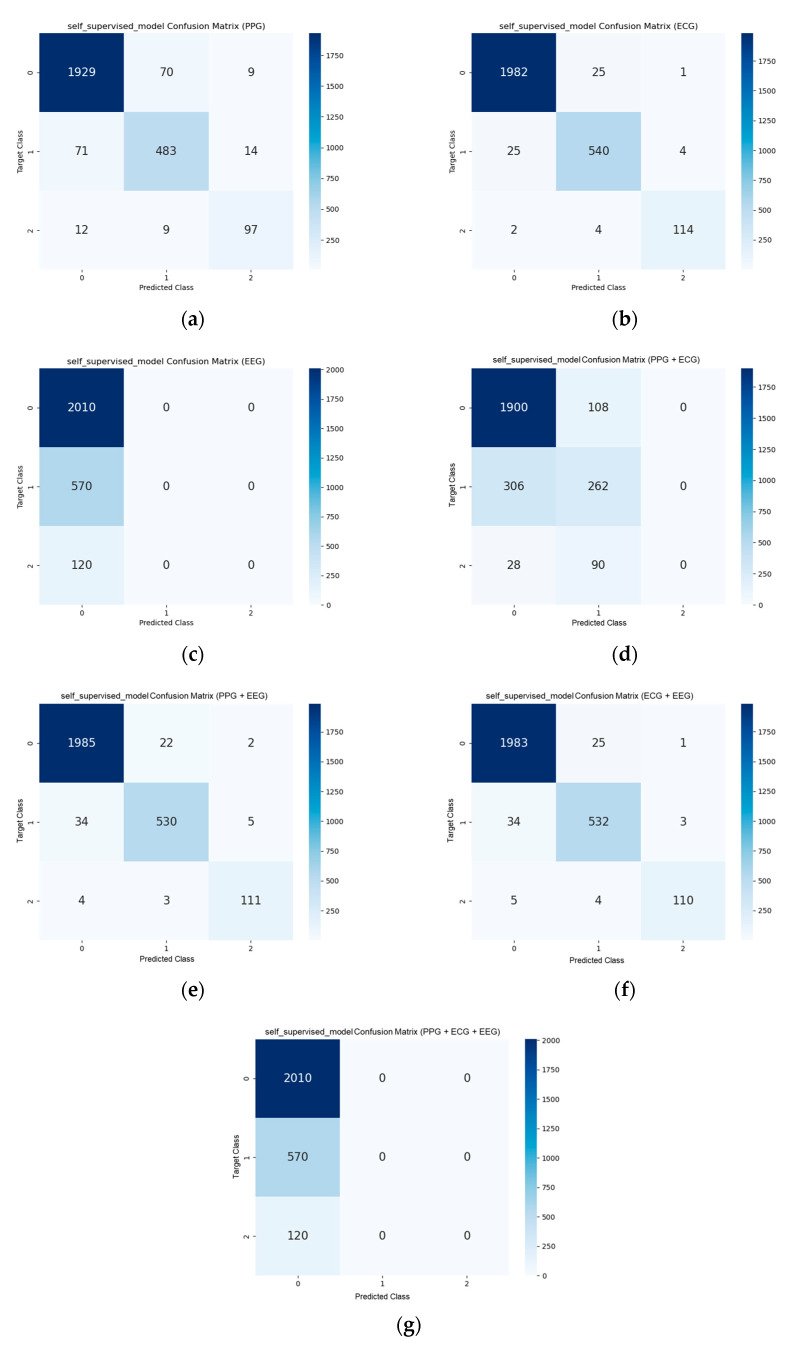
Self-supervised CNN confusion matrix for (**a**) PPG as input, (**b**) ECG as input, (**c**) EEG as input, (**d**) combined PPG and ECG as input, (**e**) combined PPG and EEG as input, (**f**) combined ECG and EEG as input, (**g**) combined PPG, ECG, and EEG as input.

The confusion matrices for StressNeXt, LRCN, and self-supervised CNN with different input signals for all scenarios and difficulty levels of Sudokus are displayed in Figure 11, Figure 12, and Figure 13, respectively. The total number of samples after data segmentation was 8100, with 6030 samples for stress level class 0, 1710 samples for class 1, and 360 samples for class 2. The confusion matrices were obtained by averaging the confusion matrix of the validation set in each fold of the 3-fold validation. It is apparent that there is more training data on class 0, and the model seems to lean towards predicting class 0 stress level. StressNeXt was unable to predict class 2 stress levels with ECG, whereas other models can effectively classify most of the class 2 stress using only ECG. The self-supervised CNN failed to distinguish other classes from class 0 with EEG or all signals combined as input and was incapable of classifying any class 2 stress with combined PPG and ECG as input.

Table 5 illustrates a performance comparison between our proposed model and previous studies on stress detection. The results indicate that our model outperforms others in terms of accuracy in scenario 3, where subjects solve Sudoku under comforting conditions. Similarly, in scenario 2, where subjects solve Sudoku with another individual monitoring, our model achieves the highest F1-score compared to previous models. Overall, our model demonstrates superior accuracy compared to Transformer [26], Random Forest [27], AdaBoost DT [17], DeepER Net [28], Artificial Neural Network [29], Deep ECGNet [30], and CNN-LSTM [31]. The comparison highlights that the effectiveness of these models may be specific to particular datasets, depending on the scenario design and experiment settings. Despite the fact that the study conducted by [12], which utilized Deep 1D-CNN, exhibited superior performance compared to our proposed work, it required more than five sensors as inputs, which may not be feasible for real-world applications. Furthermore, the evaluation of individual sensor data on the performance of our proposed model was not conducted, and the sample size only included 15 participants, which did not adequately represent the majority of human stress levels. Similarly, the study using SVM-RBF [25] demonstrated slightly higher accuracy than our proposed work. However, it is worth noting that our proposed work achieved higher accuracy (93.42%) when utilizing only EEG signals, compared to [13], which utilized PPG (80%) and GSR + PPG (86.25%) signals. Perfect comparison becomes relatively challenging as our current study did not incorporate the GSR signal, as its changes vary based on environmental conditions. In general, most studies included fewer than 20 participants, and variations in task creation and labeling methods likely contribute to performance differences. Nonetheless, our study attains the highest accuracy (98.78%) in scenario 3 and the highest F1-score (96.67%) in scenario 2, surpassing the existing work.

## 5. Conclusions

In this study, a novel experimental approach was used to evaluate stress levels in participants through the use of Sudoku puzzles as problem-solving tasks. The study utilized PPG, ECG, and EEG data to extract features and assess stress levels. Various scenarios were created using audio and video components to examine the impact of environmental stimuli on stress levels. The results indicated that noisy environments tended to cause higher stress levels, and higher difficulty level Sudoku puzzles may lead to a higher average stress level. The stress detection models used in the experiments showed high effectiveness, with the LRCN model achieving a stress level detection accuracy of 93.42% and an F1-score of 88.11% when ECG was used as an input signal. When analyzing different scenarios, the StressNeXt model demonstrated exceptional effectiveness in predicting stress levels under comforting conditions, achieving an accuracy rate of 98.78% and an F1-score of 95.39%. Conversely, the LRCN model was most effective in predicting stress levels in two scenarios, achieving accuracy rates of 95.13% and 97.96%, with F1 scores of 93.72% and 96.67%, respectively. However, the study has some limitations, such as the subjectivity of self-reporting, which can result in variations in reported stress levels. To improve the reliability of this study, more robust self-reporting methods, such as using the Self-Assessment Manikin (SAM), can be employed to validate the accuracy and reliability of stress level assessment. In addition, this study aims to explore the potential inclusion of other physiological signals, such as GSR or respiratory rate, to examine their effectiveness in stress detection. Furthermore, the study plans to incorporate another dataset containing diverse scenarios and environments to validate the performance of the proposed deep learning models.

## Figures and Tables

**Table 1:** Participant information. Long dash (—) means no participant.

Faculty	Sophomore	Junior	Senior	Ph.D.	Total
FOSE	2	1	2	2	7
FHSS	11	—	—	—	11
NUBS	2	3	6	1	12
Total	15	4	8	3	30

**Table 2:** Performances of various models and different data combinations.

Model Name	Data	Accuracy	F1-Score
StressNeXt	PPG	83.90%	69.04%
	ECG	85.71%	69.61%
	EEG	66.31%	39.40%
	PPG + ECG	88.22%	77.48%
	PPG + EEG	83.26%	68.35%
	ECG + EEG	90.02%	80.45%
	PPG + ECG + EEG	86.90%	74.26%
LRCN	PPG	84.27%	70.49%
	ECG	93.42%	88.11%
	EEG	80.15%	62.36%
	PPG + ECG	86.77%	74.66%
	PPG + EEG	83.89%	69.67%
	ECG + EEG	91.39%	84.31%
	PPG + ECG + EEG	84.44%	71.35%
Self-Supervised CNN	PPG	81.66%	63.98%
	ECG	90.07%	81.11%
	EEG	74.44%	28.90%
	PPG + ECG	86.05%	71.47%
	PPG + EEG	84.72%	69.90%
	ECG + EEG	90.32%	81.04%
	PPG + ECG + EEG	80.69%	52.70%

**Table 3:** Performance of various models and different combinations of data in different scenarios.

Model Name	Scenario	Data	Accuracy	F1-Score
StressNeXt	Scenario 1	PPG	84.78%	79.90%
		ECG	93.48%	90.83%
		EEG	63.39%	48.68%
		PPG + ECG	89.52%	85.44%
		PPG + EEG	80.98%	73.95%
		ECG + EEG	92.71%	90.02%
		PPG + ECG + EEG	87.88%	84.37%
	Scenario 2	PPG	86.64%	79.53%
		ECG	96.80%	94.27%
		EEG	64.81%	47.75%
		PPG + ECG	83.73%	65.20%
		PPG + EEG	84.18%	75.01%
		ECG + EEG	95.93%	91.33%
		PPG + ECG + EEG	89.37%	76.86%
	Scenario 3	PPG	95.22%	78.55%
		ECG	97.79%	91.37%
		EEG	86.86%	46.81%
		PPG + ECG	97.79%	91.62%
		PPG + EEG	94.80%	77.17%
		ECG + EEG	98.78%	95.39%
		PPG + ECG + EEG	98.29%	92.88%
LRCN	Scenario 1	PPG	81.78%	78.10%
		ECG	95.13%	93.72%
		EEG	69.93%	55.68%
		PPG + ECG	88.52%	85.41%
		PPG + EEG	81.15%	77.31%
		ECG + EEG	93.51%	91.17%
		PPG + ECG + EEG	81.51%	77.14%
	Scenario 2	PPG	82.94%	72.06%
		ECG	96.46%	93.90%
		EEG	73.35%	54.19%
		PPG + ECG	85.21%	74.37%
		PPG + EEG	83.31%	73.50%
		ECG + EEG	97.96%	96.67%
		PPG + ECG + EEG	87.84%	75.98%
	Scenario 3	PPG	95.43%	78.90%
		ECG	97.16%	93.63%
		EEG	90.42%	57.76%
		PPG + ECG	95.44%	84.94%
		PPG + EEG	92.14%	68.00%
		ECG + EEG	96.71%	90.31%
		PPG + ECG + EEG	92.00%	67.49%
Self-Supervised CNN	Scenario 1	PPG	92.06%	90.09%
		ECG	95.06%	93.01%
		EEG	63.35%	28.10%
		PPG + ECG	88.22%	82.41%
		PPG + EEG	91.08%	89.11%
		ECG + EEG	87.62%	80.42%
		PPG + ECG + EEG	90.05%	87.98%
	Scenario 2	PPG	92.95%	89.92%
		ECG	96.61%	94.54%
		EEG	69.90%	30.35%
		PPG + ECG	90.53%	79.41%
		PPG + EEG	90.08%	85.39%
		ECG + EEG	96.15%	93.09%
		PPG + ECG + EEG	88.56%	76.57%
	Scenario 3	PPG	95.48%	78.54%
		ECG	98.50%	94.66%
		EEG	89.95%	37.93%
		PPG + ECG	95.14%	77.24%
		PPG + EEG	95.80%	82.97%
		ECG + EEG	97.06%	85.73%
		PPG + ECG + EEG	94.89%	77.83%

**Table 4:** Performance of various models and different combinations of data was evaluated across different difficulty levels of Sudoku puzzles.

Model Name	Sudoku Difficulty	Data	Accuracy	F1-Score
StressNeXt	Medium	PPG	87.75%	78.58%
		ECG	90.04%	83.72%
		EEG	69.98%	44.72%
		PPG + ECG	83.79%	73.58%
		PPG + EEG	86.06%	75.56%
		ECG + EEG	87.85%	78.48%
		PPG + ECG + EEG	86.25%	76.48%
	Hard	PPG	85.36%	73.37%
		ECG	89.41%	77.91%
		EEG	67.59%	43.92%
		PPG + ECG	84.96%	70.63%
		PPG + EEG	81.80%	67.53%
		ECG + EEG	90.05%	79.82%
		PPG + ECG + EEG	84.52%	69.55%
LRCN	Medium	PPG	85.52%	74.36%
		ECG	91.56%	87.91%
		EEG	79.75%	57.72%
		PPG + ECG	84.90%	74.16%
		PPG + EEG	84.37%	70.64%
		ECG + EEG	82.83%	67.56%
		PPG + ECG + EEG	84.37%	72.07%
	Hard	PPG	82.58%	68.27%
		ECG	92.00%	83.55%
		EEG	77.14%	58.98%
		PPG + ECG	86.98%	75.84%
		PPG + EEG	81.55%	66.90%
		ECG + EEG	87.74%	75.85%
		PPG + ECG + EEG	87.10%	75.75%
Self-Supervised CNN	Medium	PPG	89.70%	81.48%
		ECG	88.71%	80.70%
		EEG	75.55%	29.64%
		PPG + ECG	87.36%	73.33%
		PPG + EEG	92.49%	84.64%
		ECG + EEG	84.92%	70.74%
		PPG + ECG + EEG	89.38%	80.53%
	Hard	PPG	91.29%	83.10%
		ECG	89.74%	81.06%
		EEG	73.34%	28.20%
		PPG + ECG	89.95%	80.51%
		PPG + EEG	84.93%	70.77%
		ECG + EEG	90.63%	82.80%
		PPG + ECG + EEG	89.81%	81.14%

**Table 5:** Comparative analysis of the performance.

Model	Accuracy	F1-Score	Input Data	Scenarios	Number of Participants
Transformer [26]	71.60%	74.20%	Raw ECG	Participants write reports for each of the two provided topics and make presentation for one of the provided topics (SWELL dataset)	25
Random Forest [27]	78.80%	88.80%	Extracted features of GSR, heart rate	Students perform multiple tasks, including sing-a-song, emails, color-word test, game, arithmetic question, social conversation, eating, homework, put hands in ice bucket	9
AdaBoost DT (3-class classification) [17]	80.34%	72.51%	Extracted features of PPG, EDA, SKT	Participants read magazines, take TSST, and watch amusing videos (WESAD dataset)	17
DeepER Net [28]	83.90%	81.00%	Extracted features of ECG and RSP	University students solve math tasks or color-word test	18
Artificial Neural Network (ANN) [29]	84.32%	78.71%	Extracted features of ACC, PPG, EDA, TEMP, RESP, EMG, and ECG	Participants read magazines, take TSST, and watch amusing videos (WESAD dataset)	17
Deep ECGNet [30]	87.39%	73.96%	Extracted features of ECG	Students take multiple tasks, including arithmetic problems, color-word test, interview	30
CNN-LSTM Network [31]	92.80%	94.56%	Raw ECG, vehicle dynamic data, environmental parameters	Participants drive on a simulator with different scenarios, including urban, highway, city	17
Multi-layer Perceptron [24]	93.64%	92.44%	Raw PPG, EDA, SKT	Participants read magazines, take TSST, and watch amusing videos (WESAD dataset)	17
SVM-RBF [25]	96.25%	96.00%	Extracted features of PPG, GSR, EEG	Participants prepare a talk and speak in front of real audience	40
Deep 1D-CNN [12]	97.48%	96.82%	ECG, EDA, EMG, RESP, TEMP, TEMP, ACC	Participants watched a series of videos	15
Proposed model (general)	93.42%	88.11%	ECG + EEG	Students solve Sudoku puzzles under different distractions, including noisy environment, another individual monitoring, comforting conditions	30
Proposed model (scenario 1)	95.13%	93.72%			
Proposed model (scenario 2)	97.76%	96.67%			
Proposed model (scenario 3)	98.78%	95.39%			

## Data Availability

Data available upon request due to ethical restrictions.
